# A Literature Review Assessing Whether the Use of Non-steroidal Anti-inflammatory Drugs (NSAIDs) Increases the Risk of Cardiovascular Events

**DOI:** 10.7759/cureus.92361

**Published:** 2025-09-15

**Authors:** Ammar Ahsan, Muhammad Arham Sahu

**Affiliations:** 1 Trauma and Orthopedics, Shrewsbury and Telford Hospital NHS Trust, Telford, GBR; 2 Trauma and Orthopedics, Hereford County Hospital, Wye Valley NHS Trust, Hereford, GBR

**Keywords:** adverse drug reactions, cardiovascular events, congestive cardiac failure, hypertension, non-steroidal anti-inflammatory drugs

## Abstract

Non-steroidal anti-inflammatory drugs (NSAIDs) are very useful due to their multiple properties, including analgesic, antipyretic, and anti-inflammatory effects. As a result, NSAIDs have become one of the most widely administered drugs in the world. The proposed function of this drug was to act like a steroid without its harmful and common side effects. However, like any other medication, NSAIDs come with their own set of side effects, notably their gastrointestinal and cardiovascular effects. With these known side effects and its ease of availability, it is concerning, and therefore further research was required to determine if there is a significant risk of cardiovascular events associated with NSAIDs. Guideline searches were performed using the following databases: National Institute for Health and Care Excellence (NICE), TRIP, SIGN, and AHRQ, which produced 22 results; however, after screening, only one guideline was included in this review. A literature search for systematic reviews was conducted using the following databases: MEDLINE, Cochrane, and PubMed, which yielded 711 results. However, after full screening, only three systematic reviews were included.

The National Institute for Health and Care Excellence (NICE) guidelines are a source of evidence-based recommendations made for healthcare professionals in the diagnosis and management of their patients. The NICE guideline focusing on NSAIDs provides advice regarding the prescription of NSAIDs, including contraindications, dosage, and mechanism of action. Three systematic reviews assessed NSAIDs and their cardiovascular effects. All three systematic reviews found an association between NSAIDs and their cardiovascular effects with varying degrees of strength. In conclusion, this review demonstrates evidence of the cardiovascular side effects related to the use of NSAIDs and raises questions about an increase in events, such as stroke, myocardial infarction, and hypertension. Evaluating the systematic reviews, it was essential to determine whether there was a statistically significant risk of cardiovascular events. All three papers suggested a linked increase in cardiovascular events; however, further research is required in order to understand which specific NSAIDs cause this. As a result, guideline alterations may need to be followed.

## Introduction and background

Non-steroidal anti-inflammatory drugs (NSAIDs) are commonly used as analgesics, anti-inflammatories, and antipyretics [1]. NSAIDs are very common; millions of people worldwide decide to treat their symptoms by self-administering NSAIDs bought over the counter (OTC) [2]. Furthermore, NSAIDs are extensively prescribed, accounting for 5-10% of all drug prescriptions [3]. Fundamentally, the goal of NSAIDs is to emulate the positive benefits of corticosteroids while eliminating the undesirable side effects of corticosteroids [4]. However, since their introduction into the markets in the 1960s, research has shown adverse drug reactions with the usage of NSAIDs [5]. Many studies have associated NSAIDs with a significant risk of gastrointestinal events; however, cardiovascular events remain less thoroughly studied [1,2,4-6]. One study suggested that the risk of myocardial infarction and stroke increases from the day a patient is started on NSAIDs [7]. It is concerning the ease of availability of NSAIDs when it is reported to have such severe adverse drug reactions. Furthermore, NSAIDs are advised as first-line treatment for conditions, such as osteoarthritis, following the National Institute for Health and Care Excellence (NICE) guidelines [8]. Further evaluation is required of the cardiovascular effects of NSAIDs.

**Figure 1:**
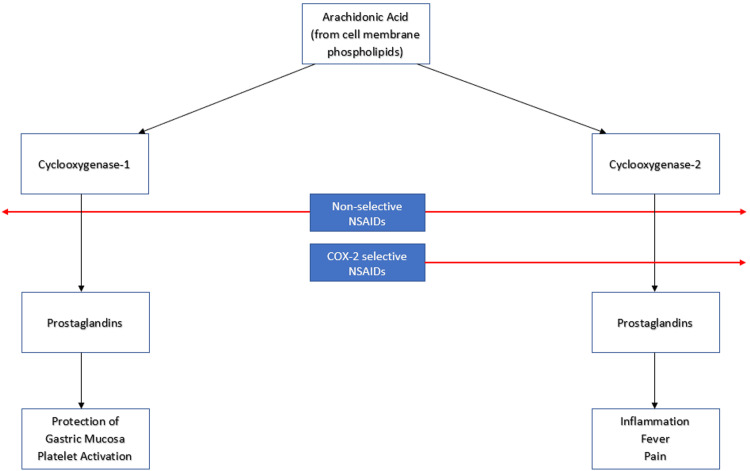
Mechanism of action of NSAIDs. NSAIDs: non-steroidal anti-inflammatory drugs; COX-2: cyclooxygenase-2

The mechanism of NSAIDs is to inhibit the enzyme cyclooxygenase (COX), which is used in the production of chemicals, such as prostaglandins [9]. As a result of the decreased prostaglandins, there is no increased temperature set point in the hypothalamus, reducing fever, and no vasodilation occurs, reducing inflammation as displayed in Figure 1 [9]. The theories suggesting an increased risk of cardiovascular events are due to the imbalance of prostaglandins and thromboxane, which is pro-thrombotic, as NSAIDs inhibit prostaglandin production, thromboxane production is unaffected, and its pro-thrombotic properties increase the risk of cardiovascular events [9]. Therefore, it was necessary to conduct a literature review of the available evidence to determine if there is a significant cardiovascular risk associated with NSAID administration.

## Review

Review question and literature searches

The research question aimed to assess whether NSAIDs increase the risk of cardiovascular events. It was defined using the "Population, Intervention, Control, Outcome (PICO)" framework (Table 1) [10]. To find all appropriate guidelines, systematic reviews, randomized controlled trials, and primary research, a comprehensive search approach was developed.

**Table 1:** Population, Intervention, Control, Outcome (PICO) framework. NSAIDs: non-steroidal anti-inflammatory drugs

Patients	Patients with NSAID exposure (prescribed for any illness)
Intervention	NSAIDs
Control	Patients not taking NSAIDs
Outcome	Cardiovascular events, such as heart failure, hypertension, and stroke

The clinical relevance of investigating whether NSAIDs increase the risk of cardiovascular events lies in the potential need to avoid their use in patients at high risk of cardiovascular disease. If the opposite is true, it provides evidence that prescription of NSAIDs is safe for patients and helps allay worries about their cardiovascular dangers.

Guideline search

The National Institute for Health and Care Excellence (NICE) evidence search was initially used as the starting point for the search for guidelines. Additional guideline searches were conducted using TRIP, SIGN, and AHRQ. The guideline inclusion criteria are presented in Table 2, and the methods are outlined in Table 3.

**Table 2:** Guideline inclusion criteria. PICO: Population, Intervention, Control, Outcome

Guideline inclusion criteria	Rationale
Guidelines must be current and published after 2019	Guidelines will have a date limit and be revised often. Therefore, it is appropriate to avoid using any old recommendations since they may no longer be therapeutically available, as they may be based on out-of-date research.
Must completely adhere to the PICO framework	This makes sure the guideline is suitable for critical appraisal.

**Table 3:** Guideline search methodology. NICE: National Institute for Health and Care Excellence; TRIP: Turning Research Into Practice; SIGN: Scottish Intercollegiate Guidelines Network; AHRQ: Agency for Healthcare Research and Quality

Databases	Keywords	Limits applied
NICE	Non-steroidal anti-inflammatory drugs, cardiovascular events, adverse drug reactions, congestive cardiac failure, and hypertension	NICE guidelines
TRIP	Non-steroidal anti-inflammatory drugs, cardiovascular events, adverse drug reactions, congestive cardiac failure, and hypertension	UK-based guidelines
SIGN	Non-steroidal anti-inflammatory drugs, cardiovascular events, adverse drug reactions, congestive cardiac failure, and hypertension	No limits
AHRQ	Non-steroidal anti-inflammatory drugs, cardiovascular events, adverse drug reactions, congestive cardiac failure, and hypertension	No limits

Systematic review search

For the systematic review search, the databases used were Cochrane, PubMed, and MEDLINE (via Ovid). The inclusion criteria for systematic reviews are outlined in Tables 3, 4. The same keywords as the guideline search were used to maintain consistency across both searches.

**Table 4:** Systematic review inclusion criteria. PICO: Population, Intervention, Control, Outcome

Systematic review inclusion criteria	Rationale
Systematic review must be published after 2005	Out-of-date data or incorrect research methodologies may be present in older publications. Moreover, data from populations from earlier research may no longer be generalizable to the current population due to the typical shift in behaviors and attitudes in a population.
Must completely adhere to PICO framework	This makes sure the systematic review is suitable for critical appraisal.

Review findings

Twenty-two results were produced by searching for guidelines on NICE, TRIP, SIGN, and AHRQ. Moving on to processing these findings via title/abstract screening, 20 were removed because they did not pertain specifically to NSAIDs or their cardiovascular risk. Following title/abstract screening, one guideline was eliminated during full guidance screening because it failed to look at the relationship between NSAID prescription and cardiovascular events. This resulted in only one guideline, which adhered to the guideline inclusion criteria, i.e., NSAIDs-prescribing issues.

**Figure 2:**
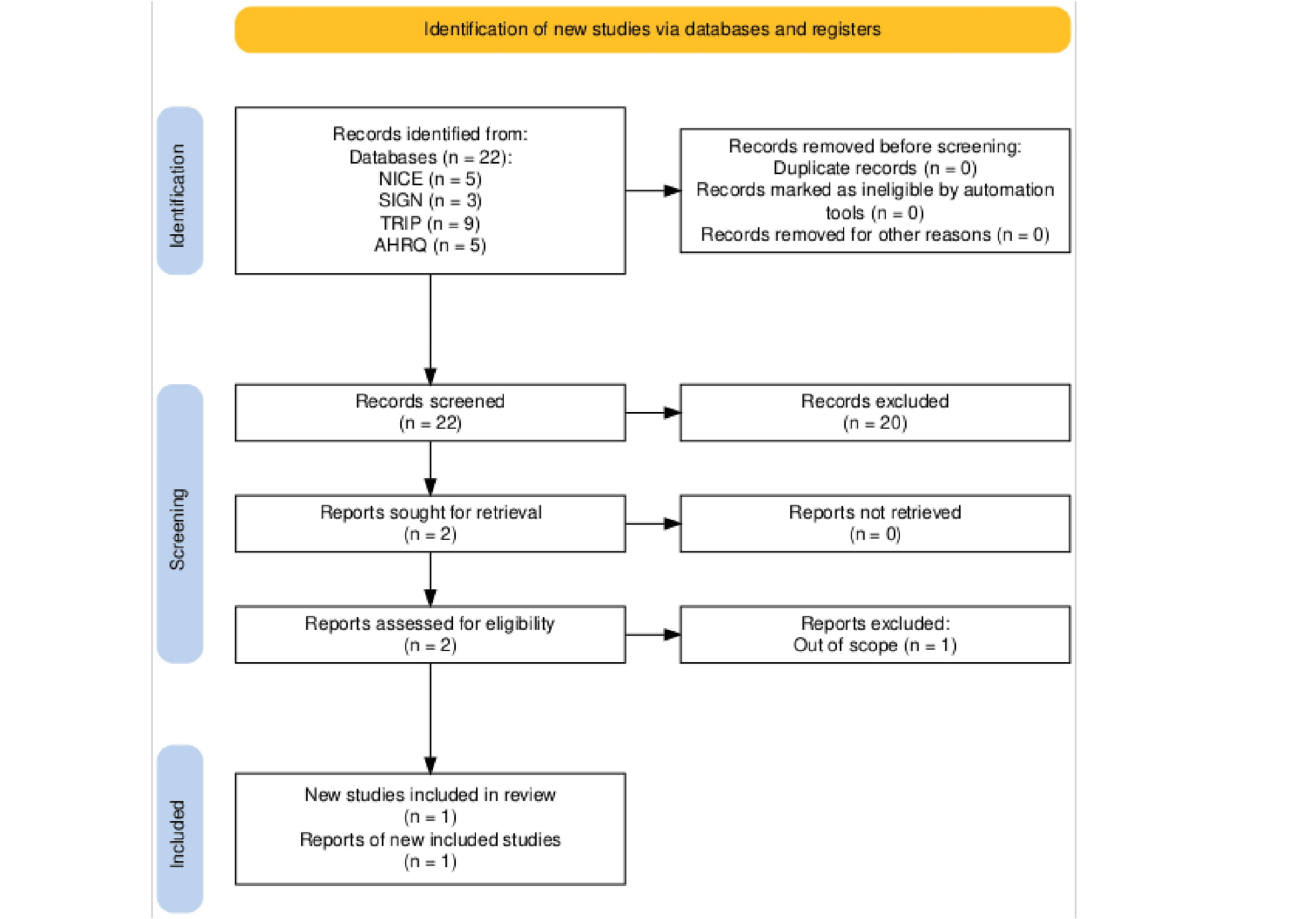
PRISMA flow diagram (guideline search). PRISMA: Preferred Reporting Items for Systematic Reviews and Meta-analyses; NICE: National Institute for Health and Care Excellence; TRIP: Turning Research Into Practice; SIGN: Scottish Intercollegiate Guidelines Network; AHRQ: Agency for Healthcare Research and Quality

**Figure 3:**
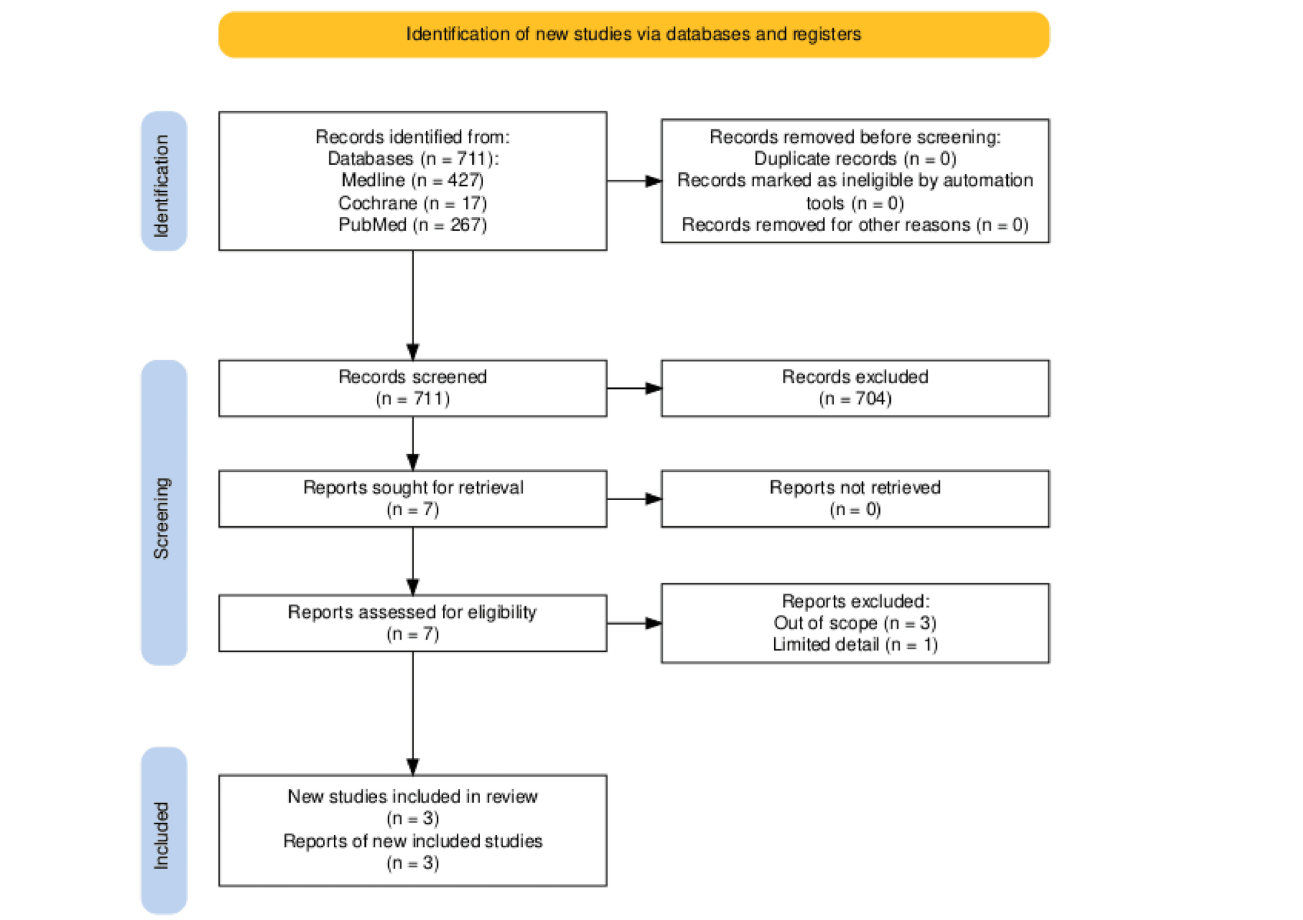
PRISMA flow diagram (systematic review search). PRISMA: Preferred Reporting Items for Systematic Reviews and Meta-Analyses

A total of 711 results were retrieved from the systematic review search. Of the 427 MEDLINE results, 423 were excluded after title and abstract screening, and the remainder of the articles were eliminated after full article screening for a variety of reasons, including using the incorrect intervention, incorrect comparison group, and incorrect outcome; general adverse drug reactions rather than cardiovascular events, thus failing to follow the PICO framework. During title/abstract screening, all 17 Cochrane findings were disregarded for not examining NSAIDs and their cardiovascular risks. Individual title/abstract screening resulted in the dismissal of 262 of 267 PubMed articles; the remaining papers underwent full article screening, which led to the elimination of two more articles, leaving three systematic reviews suitable for critical appraisal. Figures 2, 3 are flowcharts that summarize the entire process [11].

Evidence summary

The NICE guidelines present evidence-based healthcare recommendations in England to assist medical practitioners in patient diagnosis and management [12]. The guidelines cover various aspects of treatment and population-specific services. The NICE guideline titled “NSAIDs - prescribing issues” aims to ensure the appropriate and compliant prescribing of NSAIDs [13]. It advises against NSAIDs for patients with severe heart failure and ischemic heart disease [13]. The guideline covers adverse drug reactions, such as a high risk of gastrointestinal events and cardiovascular events in certain patients [13]. Formulation of the guideline is advised by organizations, such as the European Society of Cardiology; the addition of government-owned agencies poses a theoretical question regarding potential bias, but the presence of advisory bodies mitigates this risk [13].

To assess NSAIDs and their cardiovascular effects, three systematic reviews underwent critical appraisal. The systematic review by Varga et al. concluded that there was a correlation between NSAIDs and cardiovascular events, but there were other potential confounding factors that needed to be assessed [14]. A detailed search strategy and quality assessment of included studies were absent. In contrast, a systematic review by Pawlosky employed a detailed search strategy and evaluated different types of NSAIDs, comparing their cardiovascular effects [15]. A systematic review by White had provided a relative risk of 1.3, which was consistent among other reviews [16]. Included studies were from more economically developed countries, suggesting reproducibility in an English population [16]. All papers were analyzed individually using the Critical Appraisal Skills Programme (CASP) checklist [17]. Table 5 briefly demonstrates the characteristics of each study and how they align with the PICO framework.

**Table 5:** Characteristics of studies demonstrating alignment to PICO framework. PICO: Population, Intervention, Control, Outcome; COX: cyclooxygenase; NSAIDs: non-steroidal anti-inflammatory drugs

PICO	Varga et al.	Pawlosky	White
Patient	Patients are prescribed NSAIDs for multiple reasons, including osteoarthritis, rheumatoid arthritis, and post-cardiac surgery.	Patients are prescribed NSAIDs for multiple reasons, including osteoarthritis, rheumatoid arthritis, and chronic pain.	Patients prescribed NSAIDs for arthritis, hypertension, and no known heart failure.
Intervention	Various NSAIDs, including non-selective agents and selective COX-2 inhibitors.	Various NSAIDs, including non-selective agents and selective COX-2 inhibitors that are available in Canada.	Various NSAIDs, including non-selective agents and selective COX-2 inhibitors.
Control	Comparisons against non-use (placebo) or between different NSAIDs.	Comparisons against non-use (placebo) or between different NSAIDs.	Comparisons against non-use (placebo) or between different NSAIDs.
Outcome	Cardiovascular outcomes such as myocardial infarction, stroke, and heart failure.	Cardiovascular outcomes such as ischemic heart disease, cerebrovascular disease, and heart failure.	Cardiovascular outcomes such as heart failure, myocardial infarction, with a primary focus on hypertension.

Evidence appraisal

A systematic review by Varga et al., which summarized existing research including interventional and observational studies, concluded that there is enough evidence to suggest that there is an increased cardiovascular risk for patients taking NSAIDs [14]. However, this risk exhibits large variability based on various factors, including ethnicity, age, and pre-existing conditions [14]. The paper provided even more detail to deduce the pharmacokinetics involved, offering an explanation for the increased cardiovascular risk [14]. This review clearly adhered to the PICO framework, although there was some variability in the control of certain randomized controlled trials. The majority of included studies trialled an NSAID against a placebo; however, some randomized controlled trials tested one cohort of patients with one type of NSAID and the other cohort with a different type of NSAID [14]. An example of this is the VIGOR study, in which one group of patients received rofecoxib and the other received naproxen [14].

One concern about this study is that there is no evidence of any particular search strategy or the reasoning behind choosing certain RCTs to analyze in more depth. This makes it more difficult to provide an accurate assessment of certain types of bias that may be present. Although it is stated that there were no conflicts of interest, this rules out financial and non-financial conflicts [18]. The fact that there is no description of the search strategy involved means that it would be necessary to review each RCT mentioned individually to assess for any bias. It is very common for research papers of high quality to discuss how they attempted to reduce bias in the discussion section of the study; however, in this study, there is no discussion section present. Consequently, the absence of assessing the quality of the included studies ultimately questions the validity of the results.

The outcome of this review indicates that there is sufficient evidence to indicate there is an increased cardiovascular risk when taking NSAIDs long-term [14]. Although variability is present at the individual level, the paper alludes to an even higher risk in elderly patients and those with pre-existing cardiovascular conditions, with the highest risk seen in patients taking diuretics [14]. These results were inferred from the data reviewed in the studies, using a statistical standpoint. Referring to the PICO framework, the outcome assessed was cardiovascular events; therefore, we need to evaluate each cardiovascular event individually.

Multiple trials have found an increased risk of hospitalization for heart failure when NSAIDs were administered [14]. Two different trials yielded the same relative risk of 1.8 compared to a control of placebo (95% CI: 1.4-2.4/1.5-2.2), whereas another trial reported a relative risk of 2.1 [14]. This is statistically significant as the lower bound of the confidence interval was above the null hypothesis. A study by Page and Henry suggested that 19% of new diagnoses of heart failure were associated with NSAID usage [19]. Furthermore, when looking at increased blood pressure, the relative risk was 1.49 with a p-value of 0.04, again providing statistically significant evidence for the association of hypertension with the administration of NSAIDs. Although the validity of the results may be questioned, the precision is supported by the statistical evidence provided. Furthermore, these results have been backed up with a scientific explanation.

The NICE guidelines are recommendations to inform healthcare in England, supported by research [12]. The guidance can come in many forms, such as providing a concise and detailed summary of treatments and services suited for the population based on their circumstances. The fundamental role of the guidelines is to aid healthcare professionals in making informed decisions about the diagnosis and management of their patients [12]. However, it is imperative to critically appraise these guidelines with instruments, such as AGREE II [20].

The purpose of the NICE guideline “NSAIDs - prescribing issues” is to highlight situations in which the prescription of NSAIDs should be reconsidered or deemed inappropriate, and to ensure proper management of patients [13]. For instance, the guideline states to avoid NSAIDs in patients with severe heart failure and goes into more detail about which specific NSAIDS to avoid in moderate heart failure [13]. The guideline goes into further details regarding specific cardiovascular conditions and discusses NSAID usage in patients with a high risk of adverse gastrointestinal events [13].

The guideline is mainly based on the Medicines and Healthcare Products Regulatory Agency (MHRA) as well as other NICE guidelines for rheumatoid arthritis and low back pain, for which NSAIDs are a common first-line/second-line pharmaceutical treatment option [13,21]. Evidence of its cardiovascular effects is supported by the European Society of Cardiology (ESC). MHRA is a state-owned agency; there could potentially be bias in using findings made by companies that want to have contracts with the government to sell NSAIDs [22]. However, it is reassuring to see that the MHRA has its own independent advisory bodies, which mitigate this risk [22]. The guideline has a high rigor of development as it describes the search strategy involved and how evidence was selected. Only evidence from the highest level of the evidence hierarchy, systematic reviews, was used. Furthermore, the guideline states that there was no conflict of interest among guideline developers [13].

Another systematic review wanted to go into further detail on whether all NSAIDs caused a significant increase in cardiovascular events or only certain NSAIDs were responsible for this effect [15]. This systematic review stemmed from an NSAID called rofecoxib, which was recalled in September 2004 due to its increased risk of heart attack and stroke [23]. Unlike the other systematic review, this study described its search strategy with Medical Subject Headings (MeSH) keywords using multiple databases [15]. One limitation of the search strategy was the exclusion of all studies focusing entirely on NSAIDs not sold on the Canadian drug market. As a result, if any NSAIDs were sold in the United Kingdom but not in Canada, they would not have been included in this particular search strategy. Although a detailed search strategy was employed, there is no in-depth description of how the included studies were selected from the rest. The authors have shown no consideration of the rigor of the studies identified.

Similar to other systematic reviews, adhering to the PICO framework, the outcome assessed was cardiovascular events. In this systematic review, the primary outcome was myocardial infarction, with secondary outcomes that included hemorrhagic or ischemic stroke. A relative risk of 1.3 was the threshold that was set for increased cardiovascular risk; however, there was no explanation for why this value was chosen, which raises questions about the validity of the findings and the conclusions that may follow, leading to statistical bias [15]. This study finds that all NSAIDs were associated with an increased risk of stroke compared to a placebo. However, not all NSAIDs reached statistical significance; therefore, it may be due to chance. NSAIDs that showed a statistically significant association included ibuprofen, with a relative risk (RR) of 2.81 (95% CI: 1.00-11.60) [15]. Interestingly, the study reports that the NSAID naproxen was not associated with an increased risk of cardiovascular death; however, no statistical evidence was presented to substantiate this finding [15]. Furthermore, not all the statistical results of the included studies were presented, and no p-values were reported.

The findings can be applied to the local population. However, it is very important to consider each patient’s individual risk factors when managing on NSAIDs. It is recommended to use NSAIDs at the lowest effective dose; this is reiterated in the NICE guideline [13,15]. Nevertheless, the authors do admit that these findings are limited by the quality of data provided, and it would be difficult to comment on dosing regimens for any specific population [15]. This study is constrained by the fact that it did not examine the entire population; rather, it focused on a particular segment of the population, which may impact the generalizability of the results.

The systematic review conducted by White concluded that certain NSAIDs may induce a small increase in cardiovascular events; however, it should be noted that many patients taking NSAIDs are elderly people who inherently are more prone to conditions, such as arthritis, and therefore naturally are at a higher risk of cardiovascular events when compared to a younger population [16]. This result necessitates more investigation into the significance of age in relation to NSAID-associated cardiovascular risk in patients. Moreover, some of these patients were on regular medication, such as antihypertensives, so research will be required into drug interactions between NSAIDs and antihypertensives.

This review addressed a clearly focused question to ascertain the effect on blood pressure while taking NSAIDs. Following the PICO outline, the population reviewed in this study was mainly patients with morbidities, such as arthritis and hypertension, and the outcome was cardiovascular events, primarily increased arterial blood pressure. White reviewed many types of primary evidence, including cohort studies, case-control studies, and randomized controlled trials [16]. A double blind randomized clinical trial is the gold standard for evaluating interventions and is considered the most accurate form of hypothesis testing [24]. The random allocation aids in minimizing the risk of bias, as both known and unknown confounding factors are evenly distributed among groups [24]. Double blinding will reduce patient bias and researcher bias, as neither will be aware of who is being given the medication, thereby reducing the chance of overanalysis [24].

Similar to the other systematic reviews, no p-value was reported, although a relative risk of 1.4 (95% CI: 1.27-1.65) was provided, which is very similar to the relative risk of the other systematic reviews, thereby helping to bolster the validity of the findings across all studies. Many trials reviewed occurred in first-world countries, such as Denmark and America [16]. Due to the close similarities between the economies and standard of living in these countries and England, the findings are likely applicable to the English population.

Limitations

While the evidence suggests an associated elevated cardiovascular risk with NSAID use, it is important to appreciate the limitations involved in this review. After an extensive systematic review search, a limited number of systematic reviews were deemed suitable, which may reduce the generalizability of the findings. Searches were limited to publications written in English, and unpublished studies were not included, potentially introducing selection bias. Of the included systematic reviews, variability in the methodology was noted as one review lacked a clearly defined search strategy and the critical appraisal of included studies, which can cause potential introduction of selection and reporting bias. It is also challenging to evaluate the strength and significance of reported data due to the lack of p-values and insufficient reporting of confidence ranges. Furthermore, the heterogeneity of sample populations, different types of NSAIDs, and dosing administered additionally complicates direct comparison between NSAIDs and their cardiovascular effects, although not formally quantified. As some reviews accounted for age and comorbidities, these controls were not applied across all reviews, possibly influencing the observed outcomes. In spite of these limitations, the general consistency of findings obtained from various sources supports the idea that more research is required regarding NSAIDs and their cardiovascular effects.

## Conclusions

Despite NSAIDs playing a major role in today’s society and being commonly used to treat long-term conditions such as arthritis, there is evidence of an association between NSAID use and cardiovascular events. More consistent associations are seen with myocardial infarction and heart failure. Thus, an evaluation of all the available literature was necessary to understand if there truly is a significant increase in cardiovascular events, which would prompt a re-evaluation of the guidelines relating to NSAIDs and their use in certain conditions. All three systematic reviews reported a relationship between cardiovascular events and NSAIDs. Although each study had its own methodological limitations, there is sufficient evidence to prompt further RCTS and meta-analyses into specific NSAIDs and identifying which NSAIDs are linked with a statistically significant increased risk of cardiovascular events. In order to reduce cardiovascular risk while allowing safe prescribing of NSAIDs, guidelines should be carefully evaluated and should distinguish between different NSAIDs until new, robust data are available. Following this research, recommendations should be altered in order to offer NSAIDs to patients where there is no increased risk of cardiovascular events.
